# The validity and reliability of a biometrically accurate, photorealistic set of young adult body size scales based on 3D scans of White Europeans

**DOI:** 10.1371/journal.pone.0351658

**Published:** 2026-06-18

**Authors:** Bethany J. Ridley, Piers L. Cornelissen, Robin S. S. Kramer, Nadia Maalin, Sophie Mohamed, Martin J. Tovée

**Affiliations:** 1 Department of Psychology, Northumbria University, Newcastle upon Tyne, United Kingdom; 2 School of Psychology, Sport Science and Wellbeing, University of Lincoln, Lincoln, United Kingdom; 3 Department of Psychology and Counselling, Birmingham City University, Birmingham, United Kingdom; 4 Aberdeen Royal Infirmary, NHS Grampian, Aberdeen, United Kingdom; Seirei Christopher University, JAPAN

## Abstract

Young adults are a high-risk group for weight-related health problems but are often overlooked in weight management intervention design. Validated visual tools that accurately depict body size are needed to improve communication and intervention strategies. This study validated innovative, photo-realistic, gender-specific body size scales of young adults (aged 18), created from 3D scans to depict systematic changes in adiposity. A total of 110 young adults (aged 18–25) completed two behavioural tasks: (1) estimating the relative size of the bodies and (2) ranking them in ascending order of weight. To assess reliability, participants repeated the tasks three days later. Participants accurately estimated relative body sizes and ranked the images in line with the systematic BMI changes across the scales. Test–retest analyses showed good to excellent reliability for both tasks. The young adult body size scales are anthropometrically accurate, reliable, and valid tools for body size estimation tasks. They provide a robust resource for research, clinical communication, and intervention design focused on young adults.

## Introduction

Unhealthy body weight has become an increasingly common public health concern among young people worldwide. Growing proportions of young adults are located at the extremes of the body mass index (BMI) distribution, with both underweight and overweight associated with significant health and psychosocial risks [1]. In the UK, 32% of young adult men and 24% of young adult women aged 16–24 were classified as overweight or obese [2]. This is particularly concerning, as underweight, overweight, and obesity during adolescence and early adulthood predict adverse health and psychosocial outcomes across the lifespan [3]. Despite these risks, many young adults fail to recognise when their body weight falls outside the healthy range. This misperception can reduce engagement with prevention and intervention efforts, making this population particularly vulnerable to persistent unhealthy weight trajectories.

Both extremes of the BMI spectrum are associated with significant health risks. Underweight is linked to impaired immune function and, in young women, adverse fertility and pregnancy outcomes [4]. Conversely, overweight and obesity increase the risk of a wide range of non-communicable diseases, including cancers, cardiovascular disease, and type 2 diabetes, as well as mental health outcomes such as depression, anxiety, and low self-esteem [5,6]. From a young person’s perspective, the psychosocial consequences of unhealthy weight can be profound, encompassing social isolation, bullying, body dissatisfaction, and reduced academic performance [7].

Despite these risks, young adults remain a relatively neglected group in the design of weight management interventions, which have traditionally focused on children or older adults. Effective prevention and intervention strategies require tools that are both developmentally appropriate and engaging for young people. Validated visual resources that accurately depict changes in body size and shape may provide an important means of improving communication, education, and intervention delivery.

### The importance of weight management interventions in a young adult population

Although advances have been made in developing weight management interventions for children, adolescents and women, young adults remain a high-risk group that has received relatively little attention [8]. This life stage, typically defined as ages 18–25, is associated with a series of transitions that increase vulnerability to weight gain, including enrolment in higher education [9,10], changes in relationship status [11,12], and shifts in dietary and exercise behaviours [13] Young adults also engage in behaviours that contribute to excess weight, such as high levels of alcohol consumption [14], frequent intake of fast food and sugar-sweetened beverages [15,16], and sedentary lifestyles [17].

At the same time, the prevalence of eating disorders and body image disturbance is particularly high in this age group. The transition to adulthood is the peak period of onset for eating disorders, with anorexia nervosa and bulimia nervosa most commonly emerging between ages 15 and 19 [18]. Concerningly, young adults’ treatment needs in this area are often poorly met. Compared with other age groups, they experience lower access to services, higher rates of hospital admission, greater treatment dissatisfaction, and more frequent disengagement compared to other age groups, leading to poorer clinical outcomes overall [18].

Together, these findings highlight the dual vulnerability of young adults to both unhealthy weight gain and body image disturbances, and the urgent need for interventions and measurement tools specifically designed for this developmental period.

### Misperception of young adults’ body weight

Weight misperceptions are widely reported across child and adult populations, suggesting overweight and obesity often go undetected [19]. Visual normalisation theory proposes that body weight is judged relative to local body size norms, and because larger bodies have become more common, perceptions of what constitutes a “normal” body have shifted accordingly [19]. For example, within a UK population of young adults, those who socialised with overweight peers were less accurate in visually recognising overweight and obese body weights in others [20]. Such findings demonstrate the need for resources, such as visual tools, that can support accurate recognition and communication about both underweight and overweight in young adults.

### Why are visual tools necessary?

Figure Rating Scales (FRS) have been developed for children and adults (e.g., [21–23]) to improve detection of unhealthy weight by providing standardised visual stimuli [24]. However, no validated scales exist specifically for young adults, despite ongoing metabolic, hormonal, and body composition changes that distinguish them from adolescents and fully mature adults [25]. Standard adult FRS, typically representing ages 18–45, may not accurately capture body shapes in this transitional stage; Byrne and Hills [26] found adolescents produced lower body-image ratings when judging adult FRS, highlighting the need for population-specific stimuli.

Body size misperception is common among 18–25-year-olds, with many individuals with overweight or obesity classifying themselves as normal weight or selecting smaller body sizes [19,20,27]. Misperception is associated with lower perceived health risk, reduced intention to lose weight, and less engagement in health-promoting behaviours [28].

FRS provide an efficient, developmentally appropriate method to assess perception in this group, allowing measurement of body size misperception (perceived vs BMI) and body dissatisfaction (perceived vs ideal size), both linked to dieting, physical activity, weight cycling, and disordered eating‌‌ [29–31].

### Body scale ethnicity

Body composition and patterns of fat deposition vary across populations due to factors such as nutrition, physical activity, socio-economic conditions, and ethnicity [32–34]. Individuals of East Asian (EA) and South Asian (SA) ancestry typically exhibit higher adiposity and lower skeletal muscle mass than White European (WE) individuals at equivalent BMI levels [32,33]. Differences are also observed in fat distribution patterns [32,35], producing corresponding variations in body shape at a given BMI [36]. These visual differences influence body-size judgements [37], indicating that ethnically specific body scales are required to accurately represent anthropometric variation.

### Study rationale

Despite the widespread use of FRS in children and adults, no validated scales exist specifically for young adults, a population characterised by unique metabolic, hormonal, and body-composition changes [25,26]. Accurately measuring both body size perception and body dissatisfaction in this group is critical, given the prevalence of misperception and its implications for engagement with health-promoting behaviours [19,20,27,28,30,31].

The present study therefore aimed to evaluate the utility of young adult–specific FRS derived from 3D body scans, restricted to White Europeans only for this study. Participants completed two tasks: a ranking task, assessing the ability to order bodies by BMI, and a visual analogue scale (VAS) task, assessing sensitivity to incremental differences in body size.

We hypothesised that:

(1) participants would correctly order the bodies according to increasing BMI, indicating that the stimulus set preserves the ordinal structure of body-size variation; and
(2) perceived body size would increase systematically with BMI in the VAS task, demonstrating reliable perceptual discrimination between adjacent BMI levels.

## Methods

### Ethics

Ethical approval was granted by the Psychology Department ethical committee at Northumbria University Psychology Department Research Ethics Committee (Ethics reference: 120.1867). The study was pre-registered with the Open Science Framework: osf.io/vma3z. This registration is for a series of validation studies for the body stimuli used in the MapMe-2 Intervention including this young adult set (see [27]). This was an online study (using Qualtrics) and participants indicated consent by clicking on a box after reading the briefing sheet which explained the experimental procedure.

### Sample size

To estimate sample size, 31 adults completed the visual analogue scale (VAS) detection task used in this study. VAS offer several pros, including speed, simplicity, high patient acceptability, and sensitivity to small changes in subjective experience [38]. They viewed images representing the 7 National Child Measurement Programme (NCMP; https://www.gov.uk/government/collections/national-child-measurement-programme) weight categories corresponding to the 2^nd^, 25^th^, 50^th^, 75^th^, 91^st^, 98^th^, and 99.6^th^ BMI centiles and indicated the body size of each image using a VAS. We calculated the smallest detectable difference in VAS scores between successive centiles (i.e., between the 98^th^ and 99.6^th^ BMI centiles) and used the mean difference score and the standard deviation of these differences to calculate Cohen’s *d*_z_. We then used G*Power [39] to estimate the sample size required to detect this difference with α = .01, and power (1 – β = 0.95) using a *t*-test for matched pairs. G*Power returned a sample size of 109 participants; therefore, we recruited until we exceeded this with complete responses.

### Participants

A total of 110 participants were recruited via Prolific for the first phase of this study between 15/09/2023 and 6/10/2023. Inclusion criteria required participants to be young adults aged 18–25, White British, residing in the UK, and fluent in written English. Only White British participants were recruited because the stimuli were based on this population, and prior research suggests that attitudes and beliefs about overweight differ across ethnic groups [40,41].

Participants were excluded if they had a current or previous diagnosis of an eating disorder to minimize the risk of psychological distress arising from judging body images or completing questionnaires on eating disorder symptomatology. To improve data quality, pre-screeners on Prolific required participants to have a 100% study approval rate and a history of completing over 100 Prolific surveys successfully. Additionally, two attention check questions were embedded within the study, allowing identification and exclusion of participants who were not attending to the task.

Participant BMI was calculated after converting self-reported weights to kilograms and heights to metres. Table 1 shows the characteristics of these participants.

**Table 1:** Participant characteristics (N = number of participants, M = mean and SD = Standard Deviation).

	Women		Men		Non-binary	
	N = 52		N = 53		N = 5	
	M	SD	M	SD	M	SD
Age (yrs)	22.81	1.65	22.85	2.03	22.40	2.41
BMI (kg.m^2^)	24.68	5.88	24.26	3.72	28.54	8.88

For the assessment of test–retest reliability, a smaller sub-sample of 50 participants randomly selected from the original cohort completed a follow-up survey three days later. Their characteristics are described in Table 2.

**Table 2:** Test-retest participant characteristics (N = number of participants, M = mean and SD = Standard Deviation).

	Women		Men	
	N = 25		N = 25	
	M	SD	M	SD
Age (yrs)	23.00	1.32	23.29	1.97
BMI (kg.m^2^)	25.46	7.76	24.50	3.67

### Stimulus generation

Maalin et al. [42] collected high-resolution 3D body shape scans and anthropometric body measures to calculate BMI and age from 221 women and 176 men, and produced a statistical mapping between these variables, separately for men and women. This modelling allows the creation of 3D computer-generated models of bodies of a specific age and BMI that substantially improve the accuracy and precision with which assessments of body size and shape can be made. We used this model to create a set of female and male bodies for 18-year-old young adults.

All scan data included in the body shape analysis were from White participants only, as discussed above, prior research demonstrates that body composition and patterns of fat deposition differ between ethnic groups (e.g., [43–45]). These differences result in distinct body shapes at equivalent BMI values, which can impact the accuracy of weight status judgments [37].

These young adult image sets were developed as part of the MapMe-2 project, an intervention targeting childhood obesity [27].The original MapMe intervention illustrated changes in size and weight for children aged 4–5 and 10–11 years and then used young adult bodies to demonstrate what a child of a particular BMI centile would look like if they continued their current weight trajectory. The current study is a behavioural validation of these young adult scales.

For each set of young adult images (one for each gender), seven weight categories were represented:

- **Underweight:** 2nd centile (clinically low weight)
- **Lower-healthy weight:** 25th centile (clinically healthy weight)
- **Mid-healthy weight:** 50th centile (clinically healthy weight)
- **Upper-healthy weight:** 75th centile (clinically healthy weight)
- **Overweight:** 91st centile (clinically overweight)
- **Lower-extremely overweight:** 98th centile (clinically obese)
- **Upper-extremely overweight:** 99.6th centile (clinically extremely obese)

These categories were chosen in alignment with cut-offs used by the National Child Measurement Programme [46]. The corresponding BMI values for each centile are presented in Table 3, and the stimulus sets are available to view and download at osf.io/vma3z.

**Table 3:** BMI values of the young adult bodies derived from the BMI centiles at the seven weight categories.

Centile Value	BMI (kg/m^2^)	
	Female	Male
2^nd^	17.40	17.50
25^th^	20.00	20.40
50^th^	21.70	22.00
75^th^	23.70	23.85
91^st^	26.10	26.10
98^th^	29.10	29.00
99.6^th^	33.05	32.90

### Procedure

The survey, along with the participant information and consent documents, was hosted online via Qualtrics. Participants first read an information form detailing the study’s purpose, procedures, rights to withdraw, and data storage and handling, before providing informed consent via Qualtrics survey which recorded and saved their agreement. This consent was given by clicking a box at the bottom of the information page. Participants were then required to confirm that they were using a laptop or desktop computer; those using a mobile phone or tablet were automatically exited from the survey to ensure stimulus presentation was consistent and appropriately scaled. Participants were asked whether they currently or had previously been diagnosed with an eating disorder. Individuals who met this criterion were excluded from participation to minimize the risk of psychological distress when judging body images or reporting their own anthropometric information.

Participants reported demographic information including age, gender (man, woman, non-binary, prefer not to say, prefer to self-describe), ethnicity (to confirm White British status), weight (in stones and pounds, kilograms, or pounds), and height (in feet and inches or centimetres). They also indicated whether they were a student (undergraduate or postgraduate) or, if not, described their current occupation.

### Body weight perception tasks

Participants then completed two body weight perception tasks for both the male and female young adult FRS sets:

1. **Ranking Task** – Participants were presented with all seven bodies from a stimulus set in random order and were asked to arrange them in ascending order of BMI centile. This was achieved using seven numbered boxes (1–7) on which participants drag-and-dropped the bodies into order.
2. **Visual Analogue Scale (VAS) Task** – Each body image was presented individually, and participants rated its size on a VAS ranging from 0 (“extremely underweight”) to 100 (“extremely overweight”).

The order of the tasks was counterbalanced across participants: half completed the ranking task first, while the other half completed the VAS task first, to control for potential order effects.

To assess test–retest reliability, a sub-sample of 50 participants from the original cohort were sent a follow-up survey via their Prolific account three days later, in which they repeated the same body weight perception tasks.

For the VAS task, we wanted to know whether participants could detect the directional differences between stimuli. To do this, we analysed how participants assigned VAS values (0–100) to stimuli. To minimize the influence of range equalising and centring biases, we normalised the VAS response data [47] per participant and stimulus type. In analysing the VAS weight data, we aimed to preserve the relative ordering of individual participants’ responses while retaining sensitivity to quantitative differences between successive BMI centiles across stimulus sex and age. Rank-based scoring would satisfy the former requirement but remove information relevant to the latter. However, using raw VAS scores risks introducing unwanted variance due to range equalisation and centring biases [47], whereby participants differ in both the span and location of the scale they employ. Such variation could obscure true differences in discriminability across BMI centiles. As a compromise consistent with psychophysical scaling practice, we normalised VAS weight responses within participant. For each participant, responses were rescaled by subtracting the minimum VAS value, dividing by the participant-specific range computed across stimulus sex and age, and multiplying by 100. This procedure preserves ordinal information while reducing inter-individual scaling artefacts, thereby improving comparability of discriminability across participants (see [48,49]). For comparison, we also include an analysis of the raw VAS data in the Supplementary Materials

## Results

We obtained complete datasets for 110 adults who self-reported being White. The datasets are available from the Open Science Framework: osf.io/vma3z.

### Validation of stimuli: Rank ordering

Table 4 is a set of confusion matrices showing patterns of match and mismatch between the rank orders of the stimuli (S), which increased in accordance with the UK90 growth reference [50], and the rank ordering reported by the participant (R). Values in cells show the percentage of stimuli in each weight category distributed amongst the 7 possible response categories. Note that there is a 14.3% chance of a correct guess. Shaded cells indicate congruent responses, i.e., the percentage of trials (calculated separately for each row) in which the participants’ chosen rank matched the required rank. These matrices are designed to illustrate whether judgement congruence was higher towards the lower, mid, or upper end of the figure weight distribution. Table 4 shows that congruence is at a high level for all weight centiles, and both image types. The lowest score (73.6% correct) was for ranking of adult women at weight category 3. A multinomial simulation of a group of 110 participants, guessing the correct rankings across 100,000 resamples, showed that a score of 30% correct for any given weight category was at the 99.99^th^ percentile of the distribution. This strongly suggests that even the worst performance by participants, 73.6%, was significantly better than guessing (i.e., 14% correct) at p < .0001.

**Table 4:** Young adults’ responses in the rank ordering task reporting percentage correct for each possible pairing of stimulus (S) and response (R). The numbers following S and R, respectively, refer to the 7 weight levels described in Table 3.

Adult		R1	R2	R3	R4	R5	R6	R7
**Women**	**S1**	93.6	5.0	0.5	0.5	0.5	0	0
	**S2**	5.5	82.3	9.1	3.2	0	0	0
	**S3**	0.5	12.3	73.6	11.4	2.3	0	0
	**S4**	0	0.9	13.6	80.9	4.5	0	0
	**S5**	0	0	3.2	4.1	90.5	1.8	0.5
	**S6**	0	0	0	0	1.8	98.2	0
	**S7**	0	0	0	0	0.5	0	99.5
**Adult**		**R1**	**R2**	**R3**	**R4**	**R5**	**R6**	**R7**
**Men**	**S1**	95.5	2.3	2.3	0	0	0	0
	**S2**	2.3	85.0	10.9	1.8	0	0	0
	**S3**	2.3	10.5	79.1	6.8	1.4	0	0
	**S4**	0	2.3	6.4	80.0	9.5	1.4	0
	**S5**	0	0	1.4	9.5	83.2	5.5	0.5
	**S6**	0	0	0	1.8	5.5	92.3	0.5
	**S7**	0	0	0	0	0	0.9	99.1

### Validation of stimuli: VAS ratings

To model the normalised VAS data and test for differences in normalised VAS scores between successive BMI centiles, we used PROC MIXED (SAS v9.4) to build a linear mixed effects model with normalised VAS as the outcome variable. As fixed effects we tested: image (i.e., adult women and adult men), BMI centile (i.e., 2, 25, 50, 75, 91, 98, 99.6), and the interaction between the two. Adult males and BMI centile 99.6 acted as the control levels for dummy coding. We added random effects at the subject level for intercept, and we used the Satterthwaite method for computing denominator degrees of freedom for the tests of fixed effects. Explanatory variables were retained in the final model if the Type III tests of fixed effects showed that they: a) were statistically significant at p < .05, and b) contributed to a statistically significant reduction in −2 log likelihood. We included participant age and BMI and the three attribution scores (genetic, over-eating, and inactivity) as covariates in the analysis.

For random effects, the model showed significant variance in intercepts across participants, Var(u_0j_) = 28.25, Z = 5.87, p < .0001. Type III tests of fixed effects showed significant effects for BMI centile (F(6,1417)= 2384.43, p < .0001), image (F(1,1417)= 225.17, p < .0001), and their two-way interaction (F(6,1417)= 27.47, p < .0001). The effect of image was attributable to images of males being assigned ~ 7.68 normalized BMI centile units on average higher than images of females (t(1417)= −15.01, p < .0001). The model parameters are shown in S1 Table of the Supplementary Materials.

S1 Table shows that the main effect of BMI centile is due to the systematic increase in VAS scores as a function of BMI centile. (Since BMI centile 99.6 was the control level in the analysis, this is reflected in S1 Table by the increasingly negative difference between the control level and the other 6 BMI centiles). The interaction between image and BMI centile is caused by the fact that VAS scores for female and male images were very similar at the lowest and highest BMI centiles. However, VAS scores were systematically lower for female than male images at intermediate BMI centiles. This model explained 90.78% of the variance in VAS scores relative to the unexplained variance in VAS scores [51,52].

**Fig 1:**
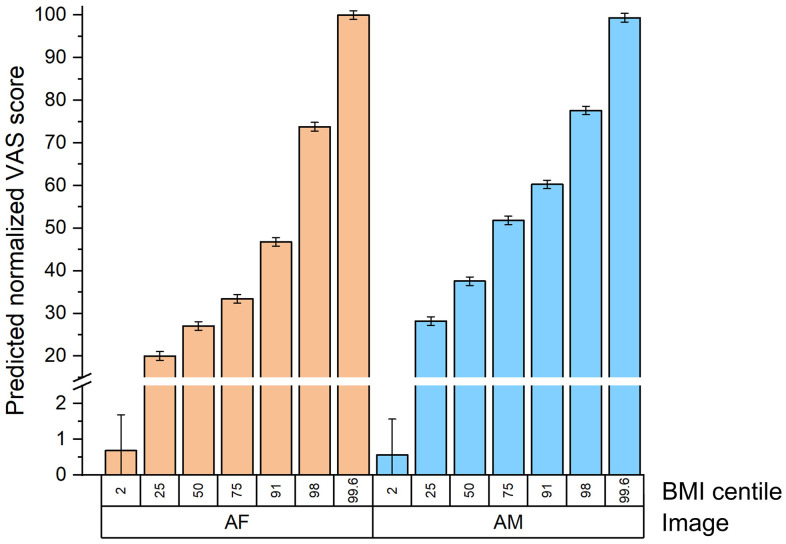
Bar charts of predicted normalised visual analogue figure size scores from the model shown inS1 Table. Error bars represent 95% CI.

Planned post-hoc comparisons, adjusted for multiple comparisons, showed that all successive increments in BMI centile, calculated separately for each image type, i.e., 2 versus 25, 25 versus 50, 50 versus 75, 75 versus 91, 91 versus 98, and 98 versus 99.6, showed statistically significant differences in normalised VAS scores at p < .05 or less. The predicted mean normalised VAS scores are shown in Fig 1.

### Test-retest

Twenty-five women and 25 men agreed to a second round of ranking and VAS measurements 3 days after the first round of measurement.

For the VAS measurements, we used a mixed-effect, two-way model for absolute agreement to compute an intra-class correlation, ICC (2, k), at each BMI centile [53,54]. The results are shown in Table 5 and suggest generally good test-retest reliability.

**Table 5:** Test-retest intra-class correlations (ICC = Intra-class correlation, CI = confidence interval, Centile = the BMI centile that a particular CGI body represents).

Centile	ICC	95% CI	Interpretation
2	0.79	0.73–0.84	Good
25	0.47	0.31–0.59	Poor
50	0.59	0.48–0.69	Moderate
75	0.75	0.68–0.81	Good
91	0.81	0.75–0.85	Good
98	0.83	0.78–0.87	Good
99.6	0.82	0.76–0.86	Good

To examine test-retest reliability for the ranking data, it is inappropriate to calculate simple intra-class correlations because the 7 locations in the rank ordering are not independent of each other, unlike the VAS data. For example, if a participant mislocates 1 image in the rank, then a second location must also contain an error: e.g., 1, 2, 3, 5, 4, 6, 7. If a participant mislocates 2 images, then 2 or 3 locations will contain an error: e.g., 1, 2, 4, 3, 5, 6, 7 or 1, 2, 4, 5, 3, 6, 7. If a participant mislocates 3 images, then between 3 and 5 locations will contain an error: e.g., 1, 2, 5, 4, 3, 6, 7 or 1, 3, 2, 4, 6, 5, 7, and so on. For these reasons we used the rankICC package in R to measure the rank intraclass correlation coefficient (rank ICC) for our ordinal data which has a 3-level cluster [55]. It serves as a nonparametric alternative to Fisher’s traditional ICC, assessing the similarity or consistency of observations within the same cluster. In addition, we applied the Fisher transformation within the rank ICC package to compute confidence intervals for the rank ICC values. These were computed separately for female (rank ICC = 0.96, 95%CI = 0.94–0.97) and male (rank ICC = 0.95, 95%CI = 0.90–0.97) images, both of which showed good test-retest reliability.

## Discussion

### Summary of findings

The present findings validate a young adult–specific FRS for both research and applied intervention contexts, providing a developmentally tailored tool to assess body size perception and dissatisfaction during a period of heightened vulnerability to misperception and unhealthy weight trajectories. We have developed a new generation of figure-rating stimuli derived from high-resolution 3D body scans. Unlike traditional FRS instruments, which typically rely on artist-drawn or digitally morphed images, the MapMe stimuli are based on anthropometrically accurate body models derived from real individuals. This approach allows systematic manipulation of BMI while preserving realistic variation in body shape and fat distribution, producing visually naturalistic stimuli that more closely reflect real human morphology.

Participants were able to reliably rank-order the stimuli and discriminate relative body size within the scales. Test–retest analyses confirmed that these judgments were consistent and reliable over time. While a data-driven approach to creating stimuli from direct anthropometric measures has been argued to confer face validity [24,56], biometric accuracy alone does not guarantee that observers can accurately perceive relative body size or discriminate differences between bodies. The present findings demonstrate that young adults can perform these tasks successfully, supporting the scales as valid and reliable tools for illustrating body size and shape in this population.

The VAS task revealed no significant gender differences in size estimation at the lowest and highest BMI centiles. However, at intermediate centiles, female bodies were systematically rated as lighter than male bodies. This pattern is consistent with the fact that absolute BMI values at these centiles are typically higher for males than females, reinforcing the biometric accuracy of the FRS measures. An alternative explanation is that these judgments may be influenced by social expectation anchoring, whereby observers assume that women are lighter than men at equivalent BMI levels.

### Young adult FRS: Implications for research and practice

The present findings highlight the potential value of the FRS as a tool for research and intervention contexts. FRS instruments are well suited to large-scale and digital interventions targeting young adults in university, workplace, and online settings [21–23]. They are brief, intuitive, and easily repeated across time points, making them suitable for monitoring perceptual and motivational changes during intervention delivery [24].

Assessing both body size misperception and body dissatisfaction is particularly important when designing obesity prevention strategies for young adults. Identifying individuals who underestimate their body size may help explain disengagement from weight-management programmes [28], while measuring dissatisfaction can help ensure interventions promoting healthy weight do not inadvertently increase psychological risk [30,31].

Furthermore, widely used health metrics such as BMI are primarily designed for either growing children or fully mature adults [29], highlighting the importance of developmentally tailored assessment tools for this transitional life stage. In both research and clinical contexts, young adult body scales may improve the accuracy of weight perception judgements, facilitate communication about weight status, and better reflect body shape changes occurring during early adulthood [25,26,36,37].

By identifying body size misperception and dissatisfaction during this sensitive developmental period, such tools may enable more precise targeting of intervention components, improve engagement among individuals at risk of obesity, and support psychologically informed approaches to early obesity prevention [28,57].

### Study strengths and limitations

A key strength of the study was the recruitment of young adults (aged 18–25) to validate FRS measures specifically designed to represent this age group. This methodological decision ensures that the scales are both developmentally appropriate and reliable for the intended population.

The presentation of stimuli at a three-quarter viewing angle is another strength. A key perceptual cue to body weight is stomach depth [58]. Previous FRS measures using front-view figures obscure this cue, making weight harder to judge [59], while profile views sacrifice ecological validity. The three-quarter view balances both concerns, providing visibility of stomach depth alongside a naturalistic presentation of the body and face.

A key strength of the present study is the isolation, as far as possible, of the perceptual component of weight status judgements, minimising contamination from attitudinal factors. This distinction is consistent with theoretical models that conceptualise body image as comprising separable perceptual and attitudinal components [60,61]. By establishing the perceptual properties of the FRS under conditions that reduce attitudinal influence, the present work provides a baseline against which deviations in future studies can be more precisely interpreted.

Critically, this enables a principled dissociation between perceptual bias (i.e., systematic misestimation of body size) and attitudinal distortion (i.e., evaluative dissatisfaction or concern). Such differentiation is essential, as these components may be differentially affected across populations [21,62]. In applied contexts, particularly when working with clinical or at-risk groups (e.g., individuals with elevated body dissatisfaction or eating pathology), this distinction allows researchers to determine whether observed effects reflect altered perceptual sensitivity or shifts in attitudinal evaluation [63,64]. Consequently, the present approach enhances interpretability by allowing perceptual and attitudinal contributions to body judgement to be examined independently rather than conflated.

The inclusion of two complementary tasks also strengthened the study design. The VAS task required participants to estimate body size individually, while the ranking task required ordering of multiple bodies. Prior research has often relied on ordered arrays, which can artificially inflate reliability because participants may recall previous choices [65], or because judgments differ depending on whether stimuli are presented in ordered or randomised arrays [66]. By using both tasks and randomised presentation, the present study reduced such biases and provided a more robust test of participants’ perceptual accuracy.

Nonetheless, the study has limitations. The scales were derived from 3D body scans of White British young adults, limiting their applicability to more ethnically diverse populations. Prior research has demonstrated differences in body composition between ethnic groups at ages 17–18, including higher percentage body fat in South Asian females and lower percentage body fat in African-Caribbean males compared to White peers [67]. This suggests that these scales should primarily be used with people of White European descent and the development of ethnically diverse scales is therefore the next step to ensure wider applicability across different populations.

When body stimuli within an FRS do not match the participants being making judgements, participants often show reduced sensitivity and increased reliance on central scale categories [21,65,68]. This compression reduces variance and limits the ability to discriminate between adjacent body sizes, particularly in fine-grained judgments. The use of non-matched ethnic body stimuli can also lead to a significant mis-estimation of body size [37]. Moreover, FRS measures implicitly encode culturally specific norms of body size and shape. Participants from different cultural backgrounds may judge bodies relative to culturally salient reference groups rather than the scale’s intended norm [67,68]. Because identical FRS responses may not represent the same latent construct across ethnic groups, findings derived from White European FRS cannot be assumed to be directly comparable in ethnically diverse samples [69]. This limits both internal validity and generalisability. These limitations argue for the use of ethnically matched FRS measures, population-specific calibration, or parametrically controlled stimuli (e.g., ethnically accurate CGI bodies), alongside explicit tests of measurement invariance when cross-ethnic comparisons are unavoidable.

### Body scale reliability

Body judgements are commonly conceptualised as comprising two independent components: an attitudinal (or cognitive–evaluative) component and a perceptual component [60]. The attitudinal component reflects beliefs, values, and affective responses related to body size and shape, and influences judgements of both one’s own body and the bodies of others [31,62,63]. The choice of a 3-day test–retest interval balances two competing methodological considerations: minimising true change in a participant’s underlying attitudinal factors while reducing immediate memory or practice effects. Our body stimuli are designed to measure relatively stable perceptual mappings between body size and internal representations, rather than longer-term attitudinal change. Over intervals of one to two weeks, body image judgements, particularly in our young adult participants, may be influenced by genuine changes in mood, weight-related experiences, media exposure, or social comparison, introducing construct-relevant variance that can artificially attenuate reliability estimates [60,70].

Conversely, very short intervals (e.g., same-day or 24-hour retest) risk inflating reliability due to memory of prior responses or anchoring to previous choices [71,72]. Such effects are particularly salient in figure rating paradigms, where participants may recall or heuristically reproduce previous selections. A 3-day interval therefore represents an intermediate solution: it is sufficiently long to reduce explicit recall of specific responses, while remaining short enough to assume stability in the underlying perceptual construct. This approach is consistent with recommendations that test–retest intervals should be long enough to minimize recall but short enough to avoid substantive change in the construct being measured [73,74].

Importantly, short test–retest intervals are standard in psychophysical and perceptual judgement paradigms, such as this study, where the goal is to assess consistency of perceptual scaling rather than longitudinal change [75–78]. In this context, a 3-day interval provides a conservative estimate of reliability by limiting extraneous sources of variance unrelated to the measurement properties of the scale itself.

The overall test–retest reliability of our body stimuli was good, which is noteworthy given that the present study employed a particularly demanding test of body-size judgements. In most previous studies, figural stimuli are presented simultaneously, arranged in ascending size from left to right, and participants are asked to indicate which figure best represents their current body size and which represents their ideal body size [21]. Gardner et al. [65] have criticised this approach on the grounds that it is likely to artificially inflate test–retest reliability, as participants can readily remember which of the small number of figures they selected previously and its relative position within the array.

In contrast, the present study employed a more stringent assessment procedure, in which participants made body judgements without access to the full ordered array of figures, thereby minimising reliance on memory for stimulus position or ordinal rank. Under these conditions, good test–retest reliability provides stronger evidence for the stability of the underlying perceptual judgements rather than task-specific response strategies.

Reliability was lowest for bodies falling within the healthy BMI centile range, with a particular tendency for confusion between bodies 2 and 3. This pattern can be explained by the way in which body-shape judgements are encoded by the visual system. Although judgements of body weight or health are often assumed to be graded continuously across a series of intermediate levels, making fine-grained discriminations along such a continuum is perceptually demanding. Instead, the visual system appears to rely on a more efficient strategy based on categorical perception [79]. Bodies are initially assigned to discrete perceptual categories (such as underweight, healthy weight, or overweight) rather than being represented with equal precision across the entire BMI continuum. Categorical perception is a well-established property of many perceptual domains, including facial identity, gender, facial expression, and race [80]. Given the limited processing resources available for perceptual judgements, categorical coding provides an efficient means of allocating neural sensitivity to the most behaviourally relevant distinctions [79].

Consequently, perceptual sensitivity is enhanced at category boundaries, such as the transition between healthy and unhealthy body weight, while sensitivity within categories is comparatively reduced. This predicts greater reliability for judgements that span category boundaries (e.g., distinguishing underweight from healthy-weight bodies) than for judgements between bodies that fall within the same category. In the present study, this provides an explanation for the reduced test–retest reliability observed between bodies 2 and 3, both of which lie within the healthy-weight range, relative to judgements involving bodies that straddle categorical boundaries. However, the primary purpose of these FRS measures is to facilitate the identification of bodies with potentially unhealthy weight. Consequently, the CGI bodies are positioned within the BMI centile range to emphasize the extremes, where health concerns are most likely to arise, resulting in a relatively sparse representation of the healthy range. Therefore, minor misallocation within the healthy range does not undermine the intended function of these measures.

### Study implications

The validated young adult FRS measures have important applications in both research and clinical practice. In research, they can be used by young adults to assess their own body image and self-perception, improving accuracy by providing stimuli specific to their developmental stage [26]. In clinical practice, healthcare professionals could use the scales to facilitate communication with young adults about weight status, monitor progress during interventions, and support weight management strategies [81].

The scales also have broader utility in preventive interventions. For example, in the *MapMe* programme, parents are shown how their child’s body might appear as a young adult if they remain on the same weight trajectory [27,56]. The present findings confirm that the scales used in such interventions are valid and reliable representations of young adult bodies, strengthening their potential as an educational and motivational tool.

### Future directions

Future research should extend this validation work in several important ways. First, the development of ethnically diverse FRS measures is a priority. The systematic ethnic differences in body composition and fat distribution produce corresponding differences in external body shape at a given BMI [36]. Consequently, the use of a single, ethnically homogeneous set of body stimuli risks conflating BMI with ethnicity-specific morphological cues. Scales for each ethnicity, based on 3D scans of bodies from that ethnicity, will ensure that the stimuli more accurately capture variation in body composition and morphology for each population [37]. These body stimuli could then be used in cross-cultural validation studies to test whether judgments of body size and shape generalise across different cultural contexts. Such work could reveal cultural differences in how body weight is perceived, with implications for both research comparability and the tailoring of public health interventions.

Additionally, integration with digital platforms, such as the MapMe project, offers opportunities for further innovation. The validated scales could be adapted into interactive digital tools for use in mobile health (mHealth) applications, weight management programmes, or online interventions. This would increase accessibility and allow real-time monitoring of changes in self-perception during interventions.

Finally, longitudinal research should examine how young adults’ use of these scales relates to longer-term outcomes, including body image satisfaction, weight-related behaviours, and responsiveness to weight management interventions. Such work would help establish not only the validity of the scales but also their practical impact on health and wellbeing.

## Conclusions

This study validated a set of photorealistic, anthropometrically accurate figural scales for male and female young adults aged 18. These scales represent an important advance in the availability of developmentally appropriate tools for a population that has been largely neglected in weight management research and intervention design. They provide a valid and reliable resource for healthcare professionals and researchers to communicate, assess, and educate young adults about body size and weight, with broad applications across research, clinical practice, intervention development, and public health.

## Supporting information

S1 Table This table shows the parameters from the linear mixed effect model of normalized VAS scores. (DOCX)

S2 Table This table shows the parameters from linear mixed effects model of raw VAS scores. (DOCX)
