# Supplementary material for: The validity and reliability of a biometrically accurate, photorealistic set of young adult body size scales based on 3D scans of White Europeans
Source: PLoS One. 2026 Jun 18;21(6):e0351658. doi: 10.1371/journal.pone.0351658 (PMC13278476; doi:10.1371/journal.pone.0351658)
Supplement: S1 Table — (DOCX) [file pone.0351658.s001.docx]

**Supplementary Materials 1**

**1) Outcome from LME of normalized VAS scores**

Table S1 shows the parameters from the linear mixed effect model of normalized VAS scores. Please see main text for further details.

Table S1. Parameters from linear mixed effects model of normalized VAS scored

| **Effect** | **Image** | **BMIc** | **Estimate** | **SE** | **t Value** | **p Value** | **95% CI** |
| --- | --- | --- | --- | --- | --- | --- | --- |
|  |  |  |  |  |  |  |  |
| Intercept |  |  | 99.37 | 1.08 | 91.77 (940) | <.0001 | 97.26 – 101.50 |
| Image | F |  | 0.60 | 1.35 | 0.44 (1417) | .66 | -2.06 – 3.25 |
| Image | M |  | 0 | . | . | . | . |
| BMIc |  | 2 | -98.81 | 1.35 | -73.01 (1417) | <.0001 | -101.46 - -96.15 |
| BMIc |  | 25 | -71.16 | 1.35 | -52.58 (1417) | <.0001 | -73.82 - -68.51 |
| BMIc |  | 50 | -61.81 | 1.35 | -45.67 (1417) | <.0001 | -64.47 - -59.16 |
| BMIc |  | 75 | -47.54 | 1.35 | -35.13 (1417) | <.0001 | -50.20 - -44.89 |
| BMIc |  | 91 | -39.09 | 1.35 | -28.89 (1417) | <.0001 | -41.75 - -36.44 |
| BMIc |  | 98 | -21.75 | 1.35 | -16.07 (1417) | <.0001 | -24.40 - -19.09 |
| BMIc |  | 99.6 | 0 | . | . | . | . |
| Image × BMIc | F | 2 | -0.48 | 1.91 | -0.25 (1417) | .80 | -4.23 – 3.28 |
| Image × BMIc | F | 25 | -8.79 | 1.91 | -4.59 (1417) | <.0001 | -12.54 - -5.03 |
| Image × BMIc | F | 50 | -11.11 | 1.91 | -5.80 (1417) | <.0001 | -14.86 - -7.36 |
| Image × BMIc | F | 75 | -19.02 | 1.91 | -9.94 (1417) | <.0001 | -22.78 - -15.27 |
| Image × BMIc | F | 91 | -14.10 | 1.91 | -7.37 (1417) | <.0001 | -17.85 – 10.34 |
| Image × BMIc | F | 98 | -4.41 | 1.91 | -2.30 (1417) | .021 | -8.16 - -0.65 |
| Image × BMIc | F | 99.6 | 0 | . | . | . | . |
| Image × BMIc | M | 2 | 0 | . | . | . | . |
| Image × BMIc | M | 25 | 0 | . | . | . | . |
| Image × BMIc | M | 50 | 0 | . | . | . | . |
| Image × BMIc | M | 75 | 0 | . | . | . | . |
| Image × BMIc | M | 91 | 0 | . | . | . | . |
| Image × BMIc | M | 98 | 0 | . | . | . | . |
| Image × BMIc | M | 99.6 | 0 | . | . | . | . |
