# Supplementary material for: The validity and reliability of a biometrically accurate, photorealistic set of young adult body size scales based on 3D scans of White Europeans
Source: PLoS One. 2026 Jun 18;21(6):e0351658. doi: 10.1371/journal.pone.0351658 (PMC13278476; doi:10.1371/journal.pone.0351658)
Supplement: S2 Table — (DOCX) [file pone.0351658.s002.docx]

**Supplementary Materials 2**

**2) Outcome from LME of raw VAS scores**

For random effects, the model showed significant variance in intercepts across participants, Var(u_0j_) = 8.53, Z = 5.57, p < .0001. Type III tests of fixed effects showed significant effects for BMI centile (F(6,1417)= 1357.50, p<.0001), image (F(1,1417)= 374.33, p<.0001), and their two-way interaction (F(6,1417)= 29.90, p<.0001). The effect of image was attributable to images of females being assigned ~ 6.11 raw BMI centile units on average more than images of males (t(1417)= 19.35, p<.0001). The model parameters are shown in Table S2, below.

Table S2. Parameters from linear mixed effects model of raw VAS scores

| **Effect** | **Image** | **BMIc** | **Estimate** | **SE** | **t Value** | **p Value** | **95% CI** |
| --- | --- | --- | --- | --- | --- | --- | --- |
|  |  |  |  |  |  |  |  |
| Intercept |  |  | 73.90 | 0.65 | 113.21 (1067) | <.0001 | 72.62 - 75.19 |
| Image | F |  | 13.97 | 0.84 | 16.73 (1417) | <.0001 | 12.33 - 5.61 |
| Image | M |  | 0 | . | . | . | . |
| BMIc |  | 2 | -42.30 | 0.84 | -50.65 (1417) | <.0001 | -43.93 - -40.66 |
| BMIc |  | 25 | -29.65 | 0.84 | -35.51 (1417) | <.0001 | -31.29 - -28.02 |
| BMIc |  | 50 | -25.74 | 0.84 | -30.82 (1417) | <.0001 | -27.37 - -24.10 |
| BMIc |  | 75 | -19.77 | 0.84 | -23.68 (1417) | <.0001 | -21.41 - -18.13 |
| BMIc |  | 91 | -16.15 | 0.84 | -19.33 (1417) | <.0001 | -17.78 - -14.51 |
| BMIc |  | 98 | -9.20 | 0.84 | -11.01 (1417) | <.0001 | -10.83 - -7.56 |
| BMIc |  | 99.6 | 0 | . | . | . | . |
| Image × BMIc | F | 2 | -7.98 | 1.18 | -6.76 (1417) | <.0001 | -10.30 - -5.67 |
| Image × BMIc | F | 25 | -10.10 | 1.18 | -8.56 (1417) | <.0001 | -12.42 - -7.79 |
| Image × BMIc | F | 50 | -10.46 | 1.18 | -8.86 (1417) | <.0001 | -12.78 - -8.14 |
| Image × BMIc | F | 75 | -13.07 | 1.18 | -11.07 (1417) | <.0001 | -15.38 - -10.75 |
| Image × BMIc | F | 91 | -10.03 | 1.18 | -8.49 (1417) | <.0001 | -12.34 - -7.71 |
| Image × BMIc | F | 98 | -3.42 | 1.18 | -2.9 (1417) | .0038 | -5.74 - -1.11 |
| Image × BMIc | F | 99.6 | 0 | . | . | . | . |
| Image × BMIc | M | 2 | 0 | . | . | . | . |
| Image × BMIc | M | 25 | 0 | . | . | . | . |
| Image × BMIc | M | 50 | 0 | . | . | . | . |
| Image × BMIc | M | 75 | 0 | . | . | . | . |
| Image × BMIc | M | 91 | 0 | . | . | . | . |
| Image × BMIc | M | 98 | 0 | . | . | . | . |
| Image × BMIc | M | 99.6 | 0 | . | . | . | . |
